# Ultrasound-guided superficial cervical plexus block combined with clavipectoral fascial plane block or interscalene brachial plexus block in clavicle surgery: a single-centre, double-blind, randomized controlled trial

**DOI:** 10.1007/s10877-022-00968-1

**Published:** 2023-01-10

**Authors:** Guangmin Xu, Peng Su, Bing Cai, Yanyu Liu, Danfeng Jiang, Yanxia He, Miyi Zhou, Meng Zhang

**Affiliations:** 1 Department of Anesthesiology, Sichuan Academy of Medical Sciences & Sichuan Provincial People’s Hospital, Chengdu, 610072 China; 2 Chinese Academy of Sciences Sichuan Translational Medicine Research Hospital, Chengdu, 610072 China

**Keywords:** Clavipectoral fascial plane block, Interscalene brachial plexus block, Superficial cervical plexus block, Clavicular surgery, Postoperative analgesia

## Abstract

The sensory innervation of the clavicle is complex, and the best regional block technology for clavicular surgery has yet to be determined. The purpose of this study was to compare the application of ultrasound-guided superficial cervical plexus block combined with clavipectoral fascial plane block verses interscalene brachial plexus block in clavicular surgery. Fifty patients undergoing internal fixation surgery for elective clavicle fractures were randomly divided into two groups (n = 25 for each group): group I and group II. Superficial cervical plexus block combined with clavipectoral fascial plane block was used in group I, and superficial cervical plexus block combined with interscalene brachial plexus block was used in group II. The primary outcome measure was the duration of analgesia. The duration of analgesia in group I was significantly longer than that in group II (P < 0.05). The modified Bromage scale function score in group II was lower than that in group I (P < 0.01). There was no significant difference in the skin acupuncture pain score 30 min after block and visual analog scale (VAS) scores at 6 and 12 h after surgery. However, the 24 h VAS score in group I was lower than that in group II (P < 0.05). The incidence of diaphragmatic paralysis was significantly increased in group II (P < 0.01). Ultrasound-guided superficial cervical plexus block combined with clavipectoral fascial plane block can be used for clavicular surgery. It has a long postoperative analgesia time, can retain the motor function of the involved upper limb and does not cause hemidiaphragmatic paresis.

Clinical trial number and registry URL: Clinical Trials.gov; Trial registration number: ChiCTR2000039383; Date of registration: 25 October 2020.

## Introduction

Clavicular fracture is the most common injury in the shoulder, particularly in young men. It mainly occurs due to sports or traffic accidents, especially in the middle of the clavicle. Better functional results can be obtained by surgical treatment. General anaesthesia can be used during surgery, but there is a risk of nausea, vomiting, aspiration and laryngeal spasm during endotracheal intubation and extubation. Furthermore, the cost of anaesthesia increases the economic burden of patients. Regional anaesthesia can meet the requirements of satisfactory operation, avoid many complications in general and provide good postoperative analgesia [1–3].

It is well known that the skin above the clavicle is innervated by the supraclavicular nerve. However, sensory innervation of the clavicle itself is controversial [4]. Traditional superficial cervical plexus block (SCPB) combined with interscalene brachial plexus block (ISBP) can be used in the operation of clavicle fracture [5]. The brachial plexus consists of C5-8 and the anterior branch of the T1 spinal nerve, and the cervical plexus consists of the anterior branch of the deep cervical plexus and superficial cervical plexus. The supraclavicular nerve of the superficial cervical plexus is responsible for innervation of the skin above the clavicle, while the brachial plexus innervates [6] the deep muscle of the clavicle. The combination of SCPB and ISBP block can meet the needs of clavicle fracture surgery, but ISBP is prone to complications such as diaphragmatic paralysis caused by phrenic nerve block [7].

Therefore, many scholars have attempted to use different regional nerve block techniques to meet the needs of clavicle fracture surgery. Clavipectoral fascial plane block (CPB) is a new regional nerve block proposed by Valdés in 2017 and can be used in anaesthesia and postoperative analgesia for clavicle fracture surgery. There is a very clear relationship between the pectoral fascia, which covers the anterior surface of the pectoralis major muscle, and the investing layer of the deep cervical fascia, which envelops the sternocleidomastoid muscle. Since Valdés proposed using CPB for clavicle fracture surgery in 2017, many scholars have reported relevant cases [3, 8–10]. All the above studies have confirmed the anaesthesia effect and postoperative analgesic effect of CPB in clavicle fracture surgery, but further clinical control experiments are still needed.

Therefore, we designed this single-centre, double-blind, randomized controlled trial study. We performed clavicular surgery with ultrasound-guided SCPB combined with CPB or ISBP to explore the effects of two regional block techniques on anaesthesia, postoperative analgesia, upper limb muscle strength and adverse reactions in clavicular fracture surgery.

## Materials and methods

### Study design and participants

This is a single-centre double-blind, randomized controlled trial of 50 patients with American Society of Anaesthesiologists (ASA) I-II at our hospital (Teaching Hospital, Residency training Hospital). This study was performed in line with the principles of the Declaration of Helsinki. Hospital ethics committee approval was obtained before starting patient enrolment (No: 2020-462). Written informed consent was obtained from all participants. The trial was registered prior to patient enrolment at the Chinese Clinical Trial Registry on 25 October 2020 (ChiCTR2000039383).

The subjects were patients with unilateral clavicle fractures who underwent elective internal fixation of clavicle fractures in our hospital. The patients were randomly divided into two groups: patients with SCPB and CPB were included in group I, and patients with SCPB and ISBP were included in group II. There were 25 patients in each group. An anaesthesiologist (who did not participate in the other steps) was responsible for recruiting patients and determining random grouping with the use of random-number tables. On the day of operation, a nurse anaesthetist (who was blinded to the scope of the study) opened the envelope, determined the grouping of patients and was responsible for the preparation of regional anaesthesia drugs. The second anaesthesiologist (who was blinded to the patient group allocation) was responsible for the regional anaesthesia operation. The second nurse anaesthetist (who was blinded for patient group allocation) was responsible for evaluating the scale, recording research data and postoperative follow-up. The exclusion criteria were as follows: (1) cardio-cerebrovascular diseases (history of heart failure, poor control of hypertension, coronary heart disease and cerebrovascular history); (2) respiratory insufficiency (bilateral rib fractures, obstructive emphysema, etc.); (3) abnormal blood coagulation; (4) puncture site infection; (5) continuous use of analgesics for the last 3 months; and (6) allergy to local anaesthetics. All data were collected from Neusoft and MedicalSystem Technology.

### Study protocol

The surgical procedure was as follows: after the patient was brought to the anaesthesia preparation room, the venous channel was opened, and electrocardiogram, oxygen saturation values and blood pressure were monitored. All operations were performed under ultrasound guidance. Patients were grouped by anaesthesiologist: medical staff randomly allocated the patients into group I (SCPB and CPB) or group II (SCPB and ISBP) and placed the details of this group allocation in an envelope. Neither the patient nor the later researchers were aware of the group information. A nurse anaesthetist opened the envelope after obtaining the patient’s consent and then prepared regional anaesthesia drugs. All regional anaesthesia procedures were performed by the same anaesthesiologist.

**Fig. 1:**
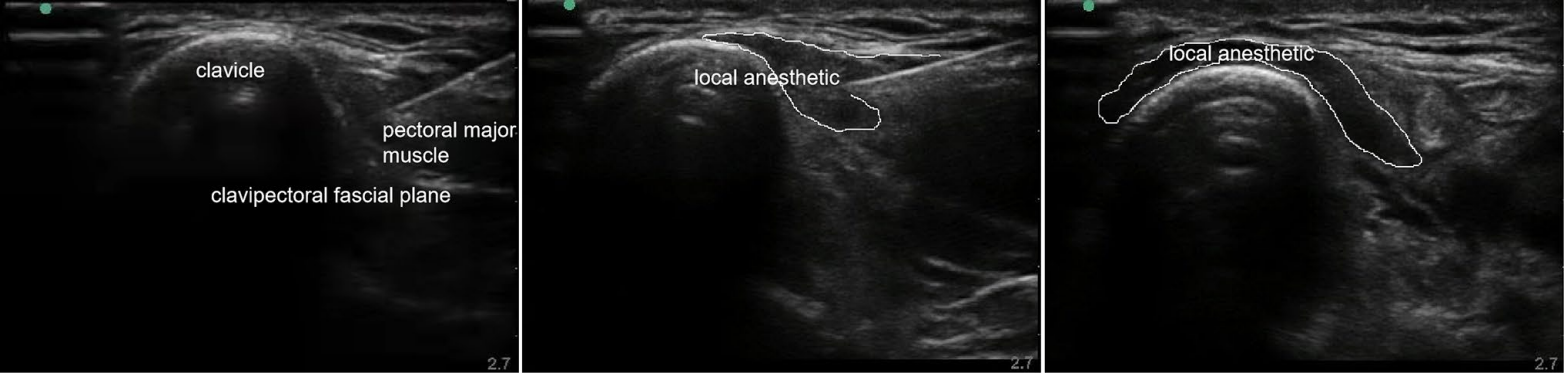
Ultrasound image of clavipectoral fascial plane block

1. Superficial cervical plexus block (SCPB): The patient was placed in a supine position with the head turned to the contralateral side for adequate exposure of the neck and the upper chest. The skin of the neck was disinfected by antiseptic solution. A linear high-frequency ultrasound probe (6–13 MHz, Sonosite) was placed at the lateral side of the neck over the midpoint of the sterno-cleido-mastoid muscle at the level of cricoid cartilage, which corresponds with the C6 transverse apophysis and its characteristic anterior tubercle. Once the muscle was identified, the probe was then moved posteriorly until the posterior tapering edge of the muscle was identified where the interscalene groove between the anterior and middle scaleni muscles was identified. Then, the superficial cervical plexus (SCP) was visualized just superficial to the prevertebral fascia overlying the interscalene groove. A five-cm block needle was then introduced from lateral to medial using the posterior-in-plane technique until its tip was placed near the SCP above the prevertebral fascia. After careful negative aspiration to exclude intravascular placement, 7 mL of 0.5% ropivacaine was deposited.
2. Clavipectoral fascial plane block (CPB): The patient was placed in a supine position with the head turned to the contralateral side, and the shoulder was padded with a small pillow. Under sterile aseptic conditions, a 6- to 13-MHz linear array probe was used for regional anaesthesia. A local anaesthetic solution of 20 mL 0.5% ropivacaine was used for regional anaesthesia. During CPB, an ultrasound probe was placed on both the inner and outer one-third of the anterior surface of the clavicle. Using the in-plane technique, a 22-gauge needle was inserted and advanced into the space between the periosteum of the clavicle and clavipectoral fascia in a caudal to cephalad direction, and a total of 20 mL of 0.5% ropivacaine was equally injected medially and laterally. The ultrasound landmarks and the in-plane needle path are shown in Fig. 1.

1. A 20-G needle was inserted using the in-plane technique from the caudal to cephalic direction.
2. Ropivacaine was injected between the periosteum of the clavicle and the surrounding fascia.
3. The spread of the local anaesthetic.

(3) Interscalene brachial plexus block (ISBP): The patient was placed in a supine position with the head turned to the contralateral side, and the shoulder was padded with a small pillow. Under sterile aseptic conditions, a 6- to 13-MHz linear array probe was used for regional anaesthesia. To perform ISBP, the high-frequency probe was positioned at the level of cricoid cartilage to visualize the brachial plexus between the anterior and middle scalene muscles. ISBP was performed with a 21-gauge short bevel needle using an in-plane technique. A total of 20 mL of 0.5% ropivacaine was injected into every 5-mL aliquot after negative aspiration, and the needle was adjusted to achieve its spread between the C5 and C6 nerve roots.
(4) A nurse anaesthetist (blinded to the patient allocation group) was responsible for evaluating the scale, recording research data and postoperative follow-up. The effect of the block was measured at 30 min in three areas: the sternoclavicular joint, midclavicle and acromioclavicular joint. If the effect was poor, the patient was changed to general anaesthesia and withdrawn from the study. At the beginning of the surgery, all patients were administered 0.05 mg/kg of midazolam.

### Outcomes measurement

The primary observation outcome was the time of first use of analgesics. Whenever postoperative pain scores were above 4, 50 mg parecoxib sodium was administered intravenously.

**Fig. 2:**
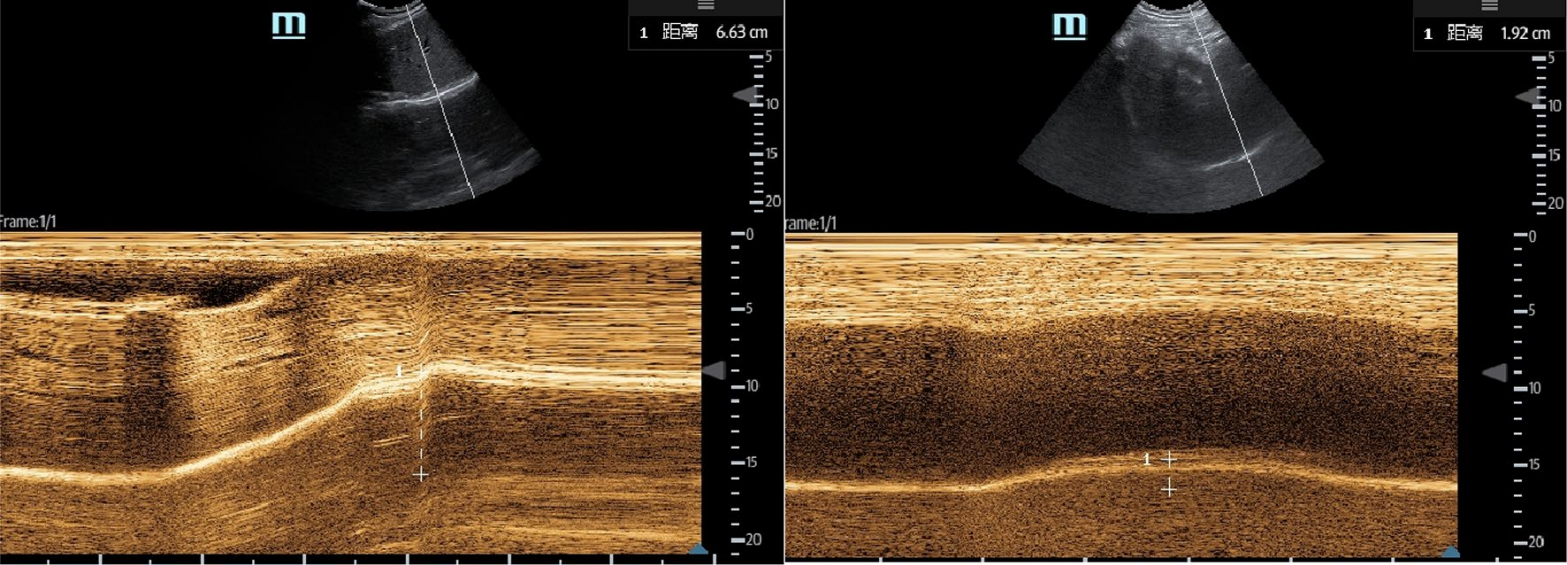
Real-time M-mode ultrasonography of the hemidiaphragm

As a secondary outcome, the effect of the block was measured at 30 min in three areas: sternoclavicular joint, midclavicular and acromioclavicular joint. Four levels were established: zero indicated no decreased sensation, one indicated decreased sensitivity to puncture, two indicated no sensitivity to puncture, and three indicated no tactile sensitivity. Values two and three were assessed as correct blocks. Modified Bromage scale (MBS) scores [11] were used to assess upper limb movement function. A score of four indicated full muscle strength in relevant muscle groups, three indicated reduced strength but the ability to move against resistance, two indicated the ability to move against gravity but not against resistance, one indicated discrete movements (trembling) of muscle groups, and zero indicated a lack of movement. The VAS scores of the patients at 6, 12 and 24 h after surgery were recorded. Diaphragmatic movement was evaluated by real-time M-mode ultrasonography of the hemidiaphragm. Patients were examined in an upright seated position. The range of diaphragmatic movement from a resting expiratory position to deep inspiration (sigh test) was recorded before and 30 min after the block (Fig. 2). Diaphragmatic movement reduction of more than 75%, no movement, or paradoxical movement was considered complete paresis. Diaphragmatic movement reduction between 25% and 75% was considered to be partial paresis, and diaphragmatic movement of less than 25% was considered no paresis. Each study subject was monitored three times before and after the block, and the average scores were used. The block-related adverse effects were recorded, including local anaesthetic systemic toxicity, nerve injury, Horner syndrome, pneumothorax, haemothorax, high epidural block and general spinal anaesthesia.

Diaphragmatic movement was evaluated by real-time M-mode ultrasonography of the hemidiaphragm (sigh test), and diaphragm movement was reduced by 71%, suggesting partial paralysis of the diaphragm.

1. Diaphragm movement is 6.63 cm from baseline measured by M-mode ultrasound before the block.
2. Diaphragm movement is 1.92 cm from baseline measured by M-mode ultrasound 30 min after the block.

### Statistical analysis

The primary outcome of this study was the time of the first postoperative analgesia. After reviewing the relevant literature [2, 3, 9], most of which were case reports, we found that the difference between the time of the first analgesia after block was 6.9 ± 5.1 h. The sample size was calculated as 0.05 with at least 20 cases in each group and a power of 0.80.

We used SPSS version 26.0 for the data analysis. Normally distributed data are expressed as the mean ± standard deviation, with two independent sample t tests. Count data are expressed as the rate (%). Grade data were analysed using the Mann–Whitney U test and are expressed as the median (M) and interquartile range (IQR). A *P value* of < 0.05 was considered statistically significant.

## Results

### Patient characteristics

**Fig. 3:**
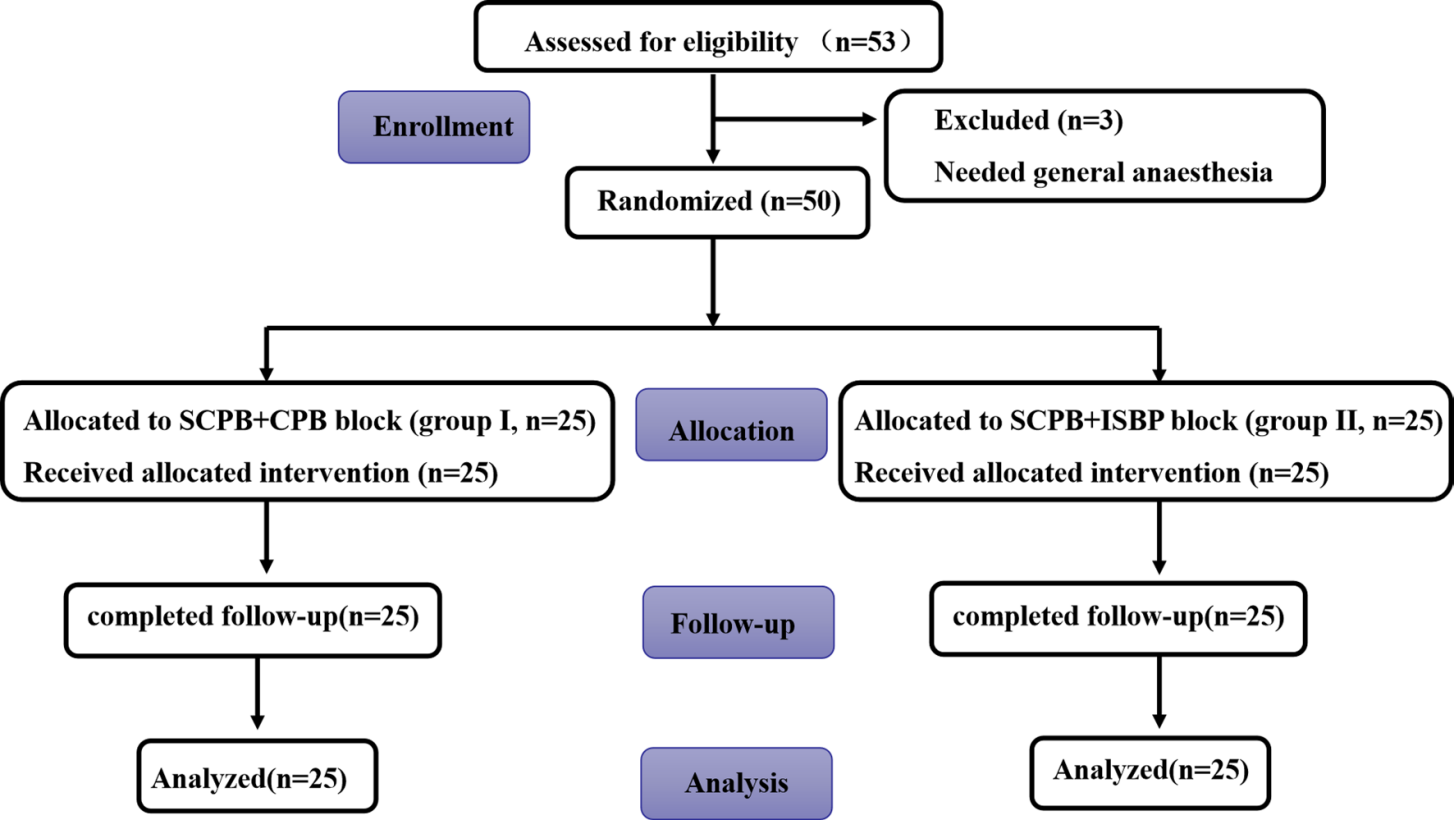
Consolidated standards of reporting trial flow diagram

From 20 December 2020 to 25 December 2021, 53 patients were eligible for enrolment and agreed to participate in the study, of whom three patients were excluded due to the need for general anaesthesia (Fig. 3). A total of 50 study subjects completed the experiment, and 25 patients were added to each group. There were no significant differences in the patient characteristics, including sex, age, height, weight, body mass index (BMI) and ASA grade, between the two groups (Table 1).

**Table 1:** Patient characteristics

Vables	Group I	Group II	*P-*value
Sex (male/female)	12/13	13/12	0.777
Age (years)	45.64 ± 11.06	45.28 ± 12.41	0.914
Height (cm)	161.76 ± 6.76	164.32 ± 7.36	0.206
Weight (kg)	58.48 ± 8.42	62.62 ± 8.84	0.096
BMI	22.25 ± 2.59	23.08 ± 2.50	0.254
ASA I/II	6/19	5/20	0.733

The values are the means ± SDs, or numbers as appropriate

*ASA* American Society of Anesthesiologists

### Time to the first use of analgesics

The first analgesic use in group I was significantly longer than that in group II (P < 0.01) (Table 2).

**Table 2:** Duration of analgesia, VAS scores, and Horner syndrome occurrence

Vables	Group I (n = 25)	Group II (n = 25)	*P*-value
Duration of analgesia (h)	20 ± 5.8	13 ± 4.3	< 0.0001^a^
VAS score after surgery			
6 h	0 (0–2)	1 (0–2)	0.264
12 h	2 (0–3)	2 (2–3)	0.080
24 h	4 (2–4)	7 (3–8)	< 0.0001^b^
Horner syndrome, n (%)	0 (0)	3 (12)	0.117^c^

Data are expressed as the mean ± standard deviation, median (interquartile range)

*VAS* visual analog scale

^a^Compared using the Student t-test

^b^Compared using the Mann–Whitney U test

^c^Compared using Fisher’s exact test

### The VAS pain scores

The VAS pain scores at 6 and 12 h were not different between the two groups; however, the VAS pain score at 24 h in group I was lower than that in group II (P < 0.01) (Table 2).

### Block score and MBS

After 30 min, there was no significant difference in the dimensions measured considering block effectiveness in the sternoclavicular joint, midclavicle and acromioclavicular joint between the two groups (P > 0.05). The MBS score in group II was lower than that in group I, and all motor function in group I was intact (P < 0.01) (Table 3).

**Table 3:** Block score and MBS [M(IQR)]

Vables	Group I	Group II	*P-*value
Sternoclavicular joint block score	3 (2–3)	2 (2–3)	P = 0.086
Midclavicle block score	3 (3–3)	3 (2–3)	P = 0.338
Acromioclavicular joint block score	3 (2–3)	3 (3–3)	P = 0.512
MBS	4 (4–4)	1 (0–2)	P < 0.0001^a^

Data are expressed as the median (interquartile range)

*MBS* Modified Bromage scale

^a^Compared using the Mann–Whitney U test

### Hemidiaphragmatic excursion during the sign test

Diaphragmatic movement was evaluated by real-time M-mode ultrasonography of the ipsilateral hemidiaphragm. Patients were examined in an upright seated position. The range of diaphragmatic movement from the resting expiratory position to deep inspiration (sigh test) was recorded before and 30 min after the block. The baseline preblock diaphragm movement was 5.9 ± 1.2 cm and 6.2 ± 1.1 cm in group I and group II, respectively, and the diaphragm movement was 5.6 ± 1.2 cm and 2.3 ± 0.9 cm in group I and group II, respectively, after 30 min of block. The reduction in diaphragmatic movement in group II was significantly less than that in group I (P < 0.01). The incidence of hemidiaphragmatic paresis in group II was 92%, but no paresis was observed in group I (Table 4).

**Table 4:** Hemidiaphragmatic excursion during the sigh test

Vables	Group I	Group II	*P-*value
Incidence of hemidiaphragmatic paresis, n (%)	0 (0)	23 (92)	< 0.0001^b^
Diaphragmatic excursions			
M-mode sigh: baseline, cm	5.9 ± 1.2	6.2 ± 1.1	0.435
M-mode sigh: 30 min, cm	5.6 ± 1.2	2.3 ± 0.9	< 0.0001^a^
Decrease in diaphragmatic excursion, (%)	5.2 ± 4.3	61.7 ± 17.9	< 0.0001^a^

Data are expressed as the mean ± standard deviation

^a^Compared using the Student *t*-test

^b^Compared using the Chi-squared test

### Adverse reaction

There were no reports of local anaesthetic systemic toxicity, nerve injury, pneumothorax, haemothorax, high epidural block, or total spinal anaesthesia. However, 3 patients in group II developed Horner syndrome (Table 2).

## Discussion

In 2017, Valdés first proposed the regional anaesthesia technique CPB at the 36th European Society of Regional Anesthesia & Pain Therapy (ESRA) Symposium. Under the guidance of ultrasound, 10–15 mL of local anaesthesia was injected between the clavipectoral fascia and the superior aspect of the clavicle. Several case studies have confirmed that CPB can be used for anaesthesia and postoperative analgesia in clavicular surgery [3, 4, 9, 10, 12]. Therefore, we used ultrasound-guided SCPB combined with CPB and compared it with the traditional SCPB combined with ISBP to observe its application for clavicular fracture surgery.

In this study, the primary observation outcome was the time of first use of analgesics. The results of our study show that the first analgesic use in group I was 20 ± 5.8 h, which was significantly longer than that in group II, indicating that SCPB combined with CPB has longer analgesia. Leurcharusmee [13] dissected 20 cadavers (a total of 40 clavicles) and showed that the clavicle, sternoclavicular and acromioclavicular joints are innervated by the subclavian, lateral pectoral, and supraclavicular nerves. We believe that CPB is an effective regional anaesthesia technique for clavicular operations since the terminal branches of many of the sensory nerves, such as the suprascapular, subclavian, lateral pectoral, and long thoracic nerves, pass through the plane between the clavipectoral fascia and the clavicle itself. In addition, local anaesthetics were injected between the clavipectoral fascia and the superior aspect of the clavicle during CPB [14], and the rate of drug absorption was slow. However, during ISBP, the rich blood vessels in the interscalene brachial plexus make drug absorption relatively fast. In addition, the time of postoperative analgesia may be related to the dose and concentration of anaesthetics. A local anaesthetic solution of 20 mL 0.5% ropivacaine was used for regional anaesthesia during CPB, and the time of first use of analgesics was consistent with the results of Yunus [2], Atalay [3] and Kukreja [9].

There was no significant difference in the effect scores in the sternoclavicular joint, midclavicle and acromioclavicular joint between the two groups 30 min after the block. The results show that the effects of the two kinds of regional anaesthesia are very good, and our results were consistent with the results of Ince [1]. However, the MBS score in group II was lower than that in group I, and all motor function in group I was retained. A researcher[8] used healthy volunteer MRI sagittal scan images after a CPB injection of a lidocaine solution marked with gadolinium. It is important to note the lack of spread through the clavipectoral fascia to posterior planes of the solution, with the subclavian artery and the brachial plexus completely free of marked solution. This shows that the subclavian artery and the brachial plexus are not surrounded by local anaesthetics after CPB. The reason why the upper limb motor function of the patient is retained is that the brachial plexus is not blocked by local anaesthetics.

Although SCPB combined with ISBP can provide sufficient analgesia in clavicle surgery, it can lead to serious adverse reactions, such as hemidiaphragmatic paresis[15–17]. To reduce the incidence of hemidiaphragmatic paresis, anaesthesiologists have conducted a series of clinical studies, including the use of ultrasound-guided visualization technology [6, 18]; different brachial plexus block technologies, even targeting specific cervical nerve roots [19]; and the use of low concentrations and large volumes of local anaesthetics [20, 21]. Although ultrasound visualization technology improves safety and patient satisfaction, diaphragmatic paralysis is still as high as 45–100%, suggesting that new regional block technology is needed to prevent hemidiaphragmatic paresis. The incidence of hemidiaphragmatic paresis in group II was 92%, but there was no paresis in group I. Our findings show that SCPB combined with CPB can reduce the incidence of hemidiaphragmatic paresis in clavicle surgery, and our result was consistent with the results of Zhuo [22] and Juliana [10], who recommended the use of SCPB combined with CPB for clavicle surgery in patients with lung disease.

This study has several limitations. First, clavicle fractures are divided into proximal, middle and distal fractures according to the fracture site and are nondisplaced, displaced and comminuted. In this study, the effects of different fracture sites and fracture types were ignored. Second, since clavicle fractures are often common in young and middle adults, most of the cases included were nonelderly patients with good physical function. Therefore, its clinical application in elderly or critically ill patients is worthy of further exploration. Third, this study found that most patients prefer not to stay awake during surgery. They believed that even if they did not feel pain during the operation, they still felt anxious and afraid. Therefore, we suggest that some sedative drugs, such as dexmedetomidine, should be introduced into clinical practice to increase the comfort of patients.

## Conclusion

In conclusion, the effects of the block measured at 30 min of both regional anaesthesia were very satisfactory. Compared with ultrasound-guided SCPB and ISBP, SCPB combined with CPB for clavicular operation has longer postoperative analgesia, better preserves the motor function of the upper limbs and avoids the incidence of diaphragmatic paralysis. Therefore, this technique is suitable for application in clinical practice.

## Data Availability

The data that support the findings of this study can be obtained from the corresponding author upon reasonable request.
